# Proximale Varisationsosteotomie des Femurs beim Morbus Perthes

**DOI:** 10.1007/s00064-022-00778-3

**Published:** 2022-08-01

**Authors:** Adam Krátký, Manuel Johannes Kraus, Andreas H. Krieg

**Affiliations:** Kinderorthopädie, Universitätskinderspital beider Basel (UKBB), Spitalstr. 33, 4056 Basel, Schweiz

**Keywords:** Hüfterkrankung, Morbus Perthes, Kind, Femurosteotomie, Korrekturwinkel, LCP-Pädiatrie-Hüftplatte, LCPD, Perthes, Pediatric hip, Femoral osteotomy, Correction angle, LCP pediatric hip plate

## Abstract

**Operationsziel:**

Die proximale femorale Varisationsosteotomie (PVO) ist eine chirurgische Technik zur Rezentrierung des Hüftkopfes, sofern es bspw. im Rahmen der ablaufenden Legg-Calvé-Perthes(LCPD)-Krankheit zur Subluxation gekommen ist.

**Indikationen:**

Bis anhin existieren keine einheitlichen Indikationskriterien für die Containment-Therapie bei LCPD-Patienten. Einzelne, für die Deformitätsentwicklung prädiktive radiologische Faktoren, Alter bei Diagnosestellung oder Symptombeginn und Klassifikationen, welche die Pathomorphologie des Femurkopfes bezogen auf die Nekrose beschreiben, können die Indikationsstellung erleichtern.

**Kontraindikationen:**

Die absolute Kontraindikation stellt die Entwicklung eines Scharniergelenkes dar (Hinge-Abduktion). Bleibt der Femurkopf in einer Abduktionsröntgenaufnahme von 20° auch in Abwesenheit einer Hinge-Abduktion dezentriert oder besteht ein Total-Kopf-Befall, ergibt sich ebenfalls eine Kontraindikation. Relativ kontraindiziert ist die PVO bei Kindern mit Beginn der Erkrankung < 6 Jahren, in der Lateral-Pillar-Klassifikation Gruppe A soiwe bei I und II nach Catterall.

**Operationstechnik:**

Lateraler Standardzugang zum proximalen Femur. Platzieren des Anteversions-K-Drahtes ventral des Schenkelhalses. Weitere K-Drähte werden parallel mithilfe von Positionierinstrumenten in den Schenkelhals eingebracht. Der optimale Bereich für die Osteotomie wird aufgesucht. Für die Vereinfachung der Manipulation des distalen Fragments und als Referenz für die Derotation werden am Femurschaft weitere K‑Drähte eingebracht. Nach Femurosteotomie erfolgt proximales Fixieren der Platte durch sukzessives Auswechseln der K‑Drähte gegen winkelstabile Schrauben. Die interfragmentäre Kompression erzeugt eine exzentrisch positionierte Kortikalisschraube im mittleren distalen Plattenloch. Die weitere distale Fixation der Platte erfolgt mit winkelstabilen Schrauben. Auswechseln der Kortikalisschraube gegen eine weitere winkelstabile Schraube. Alternativ zur hier beschriebenen winkelstabilen Technik wurde früher meist mittels Winkelplatte korrigiert.

**Weiterbehandlung:**

Mobilisation unter Abrollbelastung an 2 Gehstöcken über 6 Wochen. Röntgenkontrolle und bei genügender Knochenkonsolidation Steigerung der Belastung. Implantatentfernung nach 9 bis 12 Monaten. Rückkehr zum Sport ab 3 Monaten.

**Ergebnisse:**

Die PVO ist eine in der Behandlung von LCPD nahezu seit 60 Jahren angewendete und weltweit etablierte chirurgische Technik. Durch ein stetig wachsendes Verständnis der Grunderkrankung wird einerseits die Indikationsstellung für operative Interventionen optimiert. Andererseits tragen neue Implantate zur Verbesserung der klinisch-radiologischen Resultate und Verringerung von Komplikationen während und nach Operation bei.

## Abstract (en)

**Objective:**

The proximal femoral varus osteotomy (FVO) aims to re-centre the femoral head in the acetabular socket after prognostically unfavourable subluxation, e.g. in Legg–Calve–Perthes disease (LCPD).

**Indications:**

No unified indication criteria have been defined yet for containment therapy in LCPD. However, specific radiographic features related to deformity development, age at diagnosis or onset and classifications describing pathomorphological changes in the femoral head related to bone necrosis can support decisionmaking.

**Contraindications:**

Absolute contraindications—a hinge abducted joint; failure of femoral head reduction in the 20° abduction anteroposterior view; total epiphyseal necrosis. Relative contraindication—children < 6 years, in lateral pillar classification group A or Catteral group I and II.

**Surgical technique:**

Lateral approach to the proximal femur. Insertion of the first K‑wire to mark the anteversion of the femoral neck. Additional K‑wires are placed parallel to the first via the positioner aiming block. Lokalise the optimal postion for the osteotomy. Insertion of additional K‑wires in the distal fragment an facilitate manipulation and serve as reference for derotation. After osteotomy proximal fixation of the plate with locking screws replacing the K-wires. Insertion of a cortical screw into the middle hole to achieve optimal interfragmentary compression. Remaining locking screws are inserted and cortical screw replaced by a locking screw.

**Postoperative management:**

Mobilization with heel-touch weight-bearing on crutches for 6 weeks. Increased weightbearing after radiographic follow-up as soon as sufficient bone union is present. Implant removal after 9–12 months. Return to sports after 3 months.

**Results:**

The FVO has been used in the surgical treatment of severe LCPD for nearly 60 years and is established worldwide. Growing knowledge and consecutive optimization of the surgery indication together with the new implants contribute to improving clinical and radiological outcomes and reducing intraoperative and postoperative complications.

## Vorbemerkungen

Die Geschichte der „Legg-Calvé-Perthes disease“ (LCPD) beginnt am Anfang des 20. Jahrhunderts mit einem heute als problematisch einzustufenden Verständnis der Erkrankung. Die damals übliche mehrere Monate andauernde Bettruhe und axiale Gelenktraktion als Therapie bedeutete erhebliche psychische Folgen für das Kind und eine soziale Belastung für die Familie. Die Aggravation der sich entwickelnden Deformation durch die Dislokation der beiden Gelenkteile war bekannt. Dabei beobachteten Ärzte eine sich verschlechternde Funktion des Hüftgelenks. Die biomechanischen Überlegungen lagen auf der Hand: die ursprünglichen räumlichen Verhältnisse von Acetabulum und Femurkopf wiederherzustellen. Diese Theorie des Containments wurde Ende der 20er-Jahre beschrieben. Zur praktischen Umsetzung des konservativen Containments wurden spezielle Orthesen oder Abduktionsschienen entwickelt. Gut 50 Jahre stand eine konservative Behandlung im Vordergrund. Im Jahr 1963 wurde von Axer die erste Operationstechnik der Containment-Behandlung bei LCPD beschrieben: die proximale varisierende Femurosteotomie (PVO) [1]. Der Grund dafür waren die unbefriedigenden Resultate der konservativen Behandlung sowie die psychosozialen Folgen derselben. Die Technik erfreute sich in den nächsten Jahrzehnten zunehmender Beliebtheit und erfuhr in den folgenden Jahren verschiedene Modifikationen. Heutzutage wird die Osteotomie häufig intertrochantär durchgeführt. Die Implantate zur Fixation entwickelten sich von einfachen Platten über Klingenplatten bis zum spezifischen winkelstabilen Implantat, der sog. locking compression plate (LCP) mit prädefiniertem Varuswinkel. Durch das fortschreitende Verständnis der Indikationskriterien hat sich um die Jahrtausendwende die Position der proximalen Varisationsosteotomie gefestigt und gilt bis heute in vielen Ländern als Goldstandard. Auf alternative operative Methoden, wie bsp. die beckenseitige Osteotomie wird hier nicht weiter eingegangen.

## Operationsprinzip und -ziel

Die PVO ermöglicht eine Rezentrierung des erkrankten Femurkopfes in der Hüftgelenkpfanne, nachdem es durch die voranschreitende LCPD zu einer Dezentrierung bzw. Subluxation des Hüftkopfes gekommen ist. Die Verteilung der übertragenden Kräfte vom Bein auf das Becken wird durch die Inkongruenz der Gelenkflächen beeinträchtigt, und die Kraftvektoren verlagern sich infolge der alterierten anatomischen Verhältnisse am kranialen Acetabulumrand auf den meist betroffenen anterolateralen Epiphysenbereich. Durch die Veränderung der Stellung von Femurschaft und Schenkelhals zueinander soll das Containment des Hüftgelenkes verbessert und das fragile Areal mit dem sich neu aufbauenden Knochen aus der Hauptbelastungszone gebracht werden, um durch diese Entlastung eine optimale Heilung und Remodellierung zu ermöglichen.

## Vorteile

- In Abhängigkeit von der vorliegenden und zu überdachenden Nekrosezone bietet die Operation vielfältige Möglichkeiten, wie man den Femurkopf im Acetabulum am besten positionieren kann. Dank der Modifikation der Osteotomie kann das Containment nicht nur in der koronaren (Varisation), sondern auch in der sagittalen (Flexions‑/Extensionsosteotomie) Ebene und in der transversalen Ebene (Derotationsosteotomie) beeinflusst werden.
- Bei der Reposition des Femurkopfes während der PVO kommt es nicht zur Verschiebung des azetabulären Rotationszentrums, wie es aber beispielsweise der Fall bei der Beckenosteotomie nach Salter ist. Dadurch kann es zur Erhöhung des intraartikulären Drucks kommen und so die Perfusion des Hüftkopfes noch verringert werden [2, 3].
- Im Vergleich zu Beckenosteotomien stellt die Operation an den Chirurgen keine übermäßigen technischen Ansprüche. Der laterale Zugang zum proximalen Femur/zum Hüftgelenk zählt zu den häufig benutzten, und die Anatomie ist übersichtlich. Die risikobehafteten Strukturen (Vasa femoralia, N. femoralis, N. ischiadicus) sind genügend weit entfernt.
- Die präoperative konventionell-radiologische Bildgebung lässt eine exakte präoperative Planung inklusive des Korrekturwinkels zu und ist mit der Situation intraoperativ gut zu korrelieren.
- Das Fragmentationsstadium kann durch die PVO überwunden und verkürzt werden. Joseph beobachtete bei 34 % der Kinder nach einer Varisationsosteotomie ein Fehlen des Fragmentationsstadiums und eine geringere Deformität durch die reduzierte laterale Subluxation [4]. Eine weitere Studie konnte zwar deutlich weniger optimistische Resultate im Hinblick auf die Deformität erbringen, jedoch war auch hier der Effekt des verkürzten Verlaufs klar nachweisbar [5].

## Nachteile

- Am häufigsten wird der PVO eine Beinverkürzung vorgeworfen. Vonseiten der Operation bildet sich die Verkürzung der Extremität durch die Osteotomie, welche zuklappend („closed wedge“) und somit varisierend im Sinne einer Verringerung des CCD-Winkels (Centrum-Collum-Diaphysen-Winkel) ausgeführt werden kann. So wandert der Femurkopf tatsächlich nach distal. Diesem Effekt kann bei Kindern durch den Verzicht auf Entnahme eines Keils entgegengewirkt werden (siehe auch Abb. 14). In der Literatur wird eine Beindiskrepanz im Durchschnitt von etwa 1 cm angegeben [1, 6, 7]. Man geht davon aus, dass ein großer Teil der Verkürzung durch die operations‑/hyperämiebedingte Wachstumsstimulation ausgeglichen wird. In der initialen Phase mag die Verkürzung gar zusätzlich die Spannung über dem Hüftgelenk und so auch den Druck auf den geschädigten Femurkopf reduzieren, was für die Abheilung durchaus auch positiv bewertet werden kann. In einer Studie mit vertretbaren LCPD-Patientenzahlen wurde die Beinlängendifferenz zwischen einer Patientengruppe nach der PVO und einer Patientengruppe nach konservativer Behandlung (Schienung zwecks der Entlastung) miteinander verglichen, und es fanden sich keine statistisch signifikanten Unterschiede [8].
- Zu den relevantesten klinischen Auffälligkeiten bei LCPD zählen sowohl das positive Trendelenburg Zeichen als auch Duchenne-Hinken, welche durch die computerisierte Ganganalyse objektiviert werden konnten [9]. Durch die Operation kommt es zur Proximalisierung des Ansatzbereichs des M. gluteus medius et minimus am Trochanter major mit resultierender Verringerung des Hebelarmes derselben Muskeln und somit ebenso zur Abduktoreninsuffizienz. Die Entwicklung einer Coxa plana und die Abnahme der artikulotrochantären Distanz akzentuieren das Phänomen noch zusätzlich.
- In der Literatur wurde als eine der langfristig unerwünschten Auswirkungen der PVO die tibiofemorale Valgisierung (Genu valgum) angegeben. Die biomechanische Überlegung weist auf die Verschiebung der mechanischen Achse und Veränderungen der Belastung der distalen Fuge am Femur hin, welche zur Angulierung während des Wachstums führen könnte [10]. In weiteren Studien wurde dieser Effekt zwar bemerkbar, allerdings ebenso in den Kontrollgruppen bei Kindern, welche sich einer konservativen Behandlung unterwarfen. Am ehesten hängt das Phänomen primär mit dem natürlichen Verlauf zusammen und sollte daher nicht zur Abwägung bezüglich Operationsindikation oder -technik herangezogen werden [11, 12].
- Die Varisierung kann eine Übervertikalisierung der Wachstumsfuge verursachen.
- Kommt es zu einer Überkorrektur der Varisation, sodass nebst der Epiphyse auch Anteile des Schenkelhalses in die Hauptbelastungszone gebracht werden, so steigt aus biomechanischer Sicht das Risiko einer Fehlbelastung, welche sowohl die Heilung der Epiphyse in möglichst sphärischer Form gefährden als auch zu einem Versagen des fixierenden Implantats führen kann.

## Indikationen

- Bis anhin existieren keine einheitlichen Indikationskriterien für Containment-Therapie bei LCPD [13].

### Prognostisch ungünstige Faktoren („head at risk signs“) im konventionellen Röntgen [14–16]

- Laterale Subluxation
- „Gage-sign“: V‑förmiger Knochendefekt im lateralen Anteil der Epiphyse
- Laterale Kalzifikation an der Epiphyse vor Beginn des Reparationsstadiums
- Horizontal positionierte Wachstumsfuge am Schenkelhals
- Metaphysär erkennbare zystische Veränderungen

### Chronologische Faktoren

- *Alter*
  - Alter bei der Manifestation > 8 Jahre und zugleich in der Gruppe B oder B/C (Lateral-Pillar-Klassifikation) [17]
  - Alter bei Diagnosestellung > 6 Jahre mit Befall von > 50 % der Epiphyse [18]
- *Timing*
  - Zeitraum zwischen dem Initial- und Fragmentationsstadium (bis Stadium IIa nach Elizabethtown-Klassifikation) [19]

### Ausmaß der Nekrose

- *Modifizierte Lateral-Pillar-Klassifikation*
  - Gruppe B und ggf. Borderline Gruppe B/C im chronologischen Alter > 8 Jahre bei der Manifestation [17]
  - Gruppe C im Alter zwischen 6 und 7,9 Jahren [20]
- *Catterall-Klassifikation* [21, 22]
  - Alle Patienten mit „head at risk signs“
  - > 6 bis 7 Jahre bei Manifestation in der Gruppe II und III (26–75 % Epiphysennekrose)
  - Gruppe IV (> 76 % Nekrose) ohne schwere Femurkopfabflachung
  - Gruppe IV mit dem Alter zwischen 6 und 10 Jahren [20]

## Kontraindikationen

- *Absolute Kontraindikationen:*
  - Vorliegen eines Scharnier-Gelenks – „hinge abduction“
    Die Ursache ist eine durch eine laterale Kalzifikation am Femurkopf gebildete Erhabenheit (sog. „Bump“) mit medial davon liegender Delle. So kommt es zur mechanischen Blockierung der Gelenkbeweglichkeit bei versuchter Abduktion.
  - Lässt sich der Femurkopf in einer Abduktionsröntgenaufnahme von 20° mit oder ohne Vorliegen eines konkreten Hinges nicht in das Acetabulum reponieren, kann das operative Verfahren dies nicht ändern, und die Indikation erübrigt sich.
  - Bei einem Total-Kopf-Befall bei Kindern ab 8 Jahren sollte nach aktuellem Stand rein konservativ behandelt werden.
- *Relative Kontraindikationen:*
  - Kinder < 6 Jahren
  - Lateral-Pillar-Klassifikation Gruppe A
  - Catterall-Klassifikation Gruppe B

## Patientenaufklärung

- Beabsichtigtes Operationsziel nach Erläuterung des Krankheitsbildes und des nun eingetretenden ungünstigen Verlaufs
- Allgemeine chirurgische Risiken und Schnittführung
- Durchtrennung des Knochens und Einbringen von Fremdmaterial
- Infekt der Wunde evtl. assoziiert mit dem Fremdmaterial
- Versagen des Metalls (Plattenbruch, Schraubenlockerung, Schraubendislokation, Verlust der Korrektur), postoperative Bewegungseinschränkungen im Hinblick auf andere Bewegungsrichtungen und geplante Implantatentfernung
- Nachbehandlung: s. unten
- Die PVO ist keine kausale Therapie der LCPD, sondern die Therapie einer prognostisch ungünstigen Komplikation: der Subluxation des Femurkopfes. Diese Tatsache muss Eltern und Patienten klar erläutert werden. Die Entscheidung für die Indikation der PVO basiert auf objektiven Fakten sowie auf den Erfahrungen behandelnder Ärzte und bringt weitgehende Konsequenzen mit sich. Im klinischen Alltag müssen Kinderorthopäden im Rahmen der „Observe and wait“-Strategie zahlreiche Faktoren berücksichtigen. Nach einer LCPD-Diagnose ist es wichtig, das Vertrauen zwischen dem Chirurgen und seinem jungen Patienten bzw. seinen Eltern aufzubauen. Die Erkrankung dauert viele Jahre an. Ein unsensibles Bestehen auf einer – möglicherweise sinnvollen – chirurgischen Behandlung könnte zum Vertrauensverlust führen.
  Die Therapieentscheidung sollte daher unter aktiver Teilnahme des Patienten und seiner Angehörigen als gleichgestellte Partner getroffen werden.

## Operationsvorbereitungen

### Präoperative Röntgenaufnahmen sollten nicht älter als 3 Monate sein

1. Beckenübersicht a.-p. tiefzentriert mit hängenden Beinen sowie Beckenübersicht a.-p. in Neutralstellung der Beine hinsichtlich der Rotation und maximaler Abduktion des betroffenen Beines, so dass eine optimale Zentrierung des Hüftkopfes im Acetabulum resultiert
2. Korrekturaufnahme der betroffenen Hüfte a.-p. mit durch den Untersucher ausgedrehter femoraler Antetorsion
3. Dunnaufnahme sowie ggf. axiale Aufnahme bei geplanter Flexions‑/Extensionskorrektur

### Korrekturplanung – funktionell

- Auf beiden a.-p.-Aufnahmen wird die Femurlängsachse eingezeichnet. Da die Aufnahme in der Abduktion eine erwünschte Kopfzentrierung ergibt, ist der Winkel zwischen den beiden Längsachsen der Korrekturwinkel. Der Korrekturwinkel lässt sich auch über eine Schablone feststellen, indem man die Kontur und Längsachse des Femurs auf eine Schablone einzeichnet und diese ins Acetabulum auf dem Röntgen reponiert.
- Der Winkel zwischen den beiden Achsen (Röntgenbild und Schablone) ist der Korrekturwinkel. Der Vorteil ist der mögliche Verzicht auf die zweite Röntgenaufnahme.

### Korrekturplanung – anhand des anatomischen CCD-Winkels

**Figure 1:**
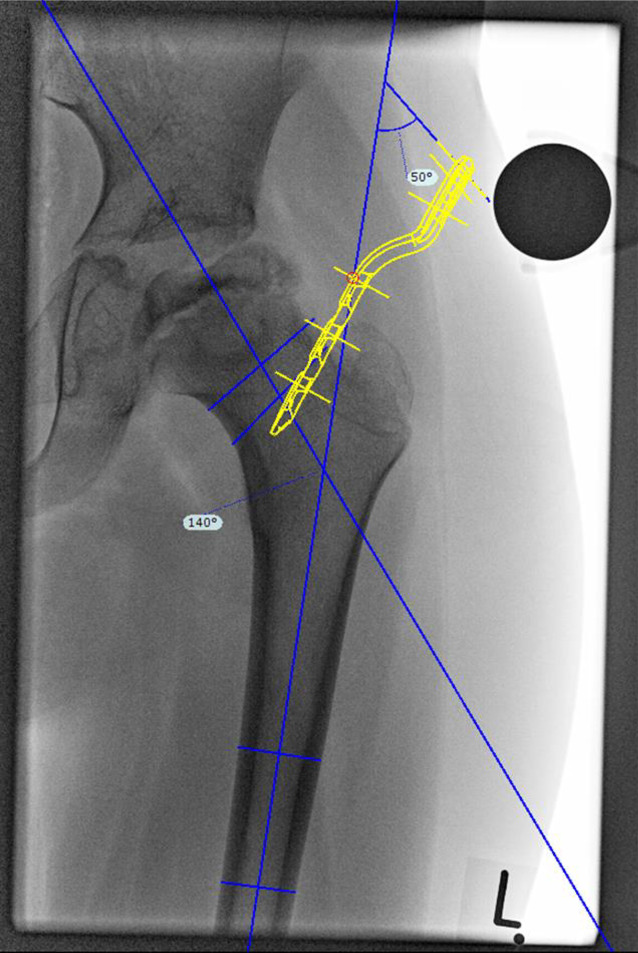

- Zuerst erfolgt das Feststellen des individuellen CCD-Winkels. Der Zielwinkel nach der Korrektur liegt normalerweise bei 120–130°. Der Unterschied zwischen dem individuellen und beabsichtigten CCD-Winkels ergibt den Korrekturwinkel.
  (Beispiel: Der individuelle CCD-Winkel der Schenkelhals- und Femurachse liegt bei 140° und der erwünschte CCD-Winkel postoperativ ist 120°. Korrekturwinkel berechnet sich aus der Differenz von beiden und ergibt 140° − 120° = 20°.)
- Von großer Bedeutung im LCP-Pädiatrie-Plattensystem ist dabei der Winkel der Platzierung der Schenkelhalsführungsdrähte bzw. später Schrauben. Dieser Winkel wird an dem justierbaren Zielgerät eingestellt und berechnet sich folgendermaßen: Zum vordefinierten Winkel der LCP-Platte wird der Korrekturwinkel addiert. Bspw. ein Plattenwinkel von 110° + 20° Korrekturwinkel ergibt 130° für die Einstellung des justierbaren Zielgerätes und somit den Platzierungswinkel für die Schenkelhalsschrauben in der koronaren Ebene (Abb. 1 und 2).

**Figure 2:**
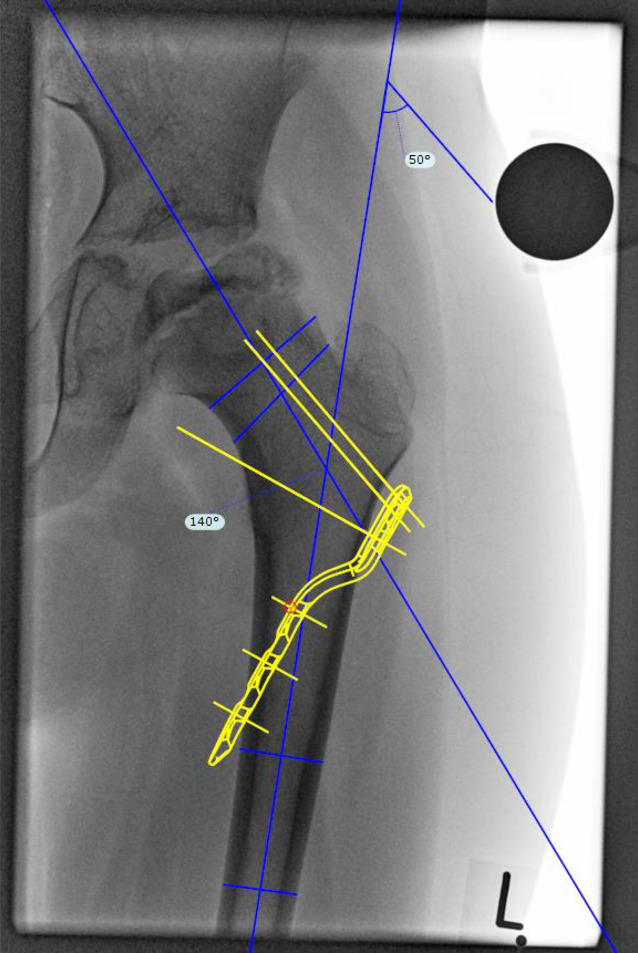

### Korrekturplanung – sagittale Ebene

- Basis einer Flexions- oder Extensionsosteotomie bildet eine axiale Röntgenaufnahme.
- Mittels einer Schablone erfolgt die präoperative Planung analog zur obigen Beschreibung.

## Instrumentarium

**Figure 3:**
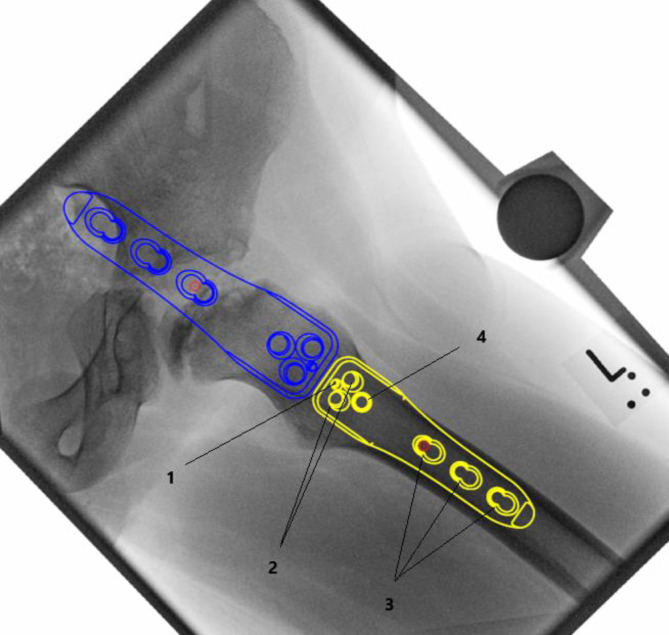

- In unserer Klinik hat sich das LCP-Pädiatrie-Platten-System bewährt. Es stehen zur Verfügung auf dem Markt 2‑ und 3‑Loch-Platten mit prädefiniertem Varisationswinkel 100° oder 110°. Der gewünschte Zielwinkel kann durch jenen Winkel, in dem die Schenkelhalsschrauben in den Schenkelhals eingebracht werden, frei gewählt werden.
  Je nach Größe der LCP-Platte unterscheidet sich der Osteotomieversatz für die Medialisierung des Femurschaftes:
  - 2,7-mm-Platte (2-Loch): 6 mm Versatz,
  - 3,5-mm-Platte (3-Loch): 8 mm Versatz,
  - 5,0-mm-Platte (3-Loch): 10 mm Versatz.
  Massgebend für die Wahl der Plattengrösse ist der Abstand der proximalsten Schrauben. Diese sollten komfortabel im Schenkelhals zu liegen kommen, ohne Risiko einer anterioren oder posterioren Perforation. Dies kann durch präoperative Planung an Dunnaufnahme oder axialem Röntgenbild beurteilt werden (Abb. 3).

## Anästhesie und Lagerung

**Figure 4:**
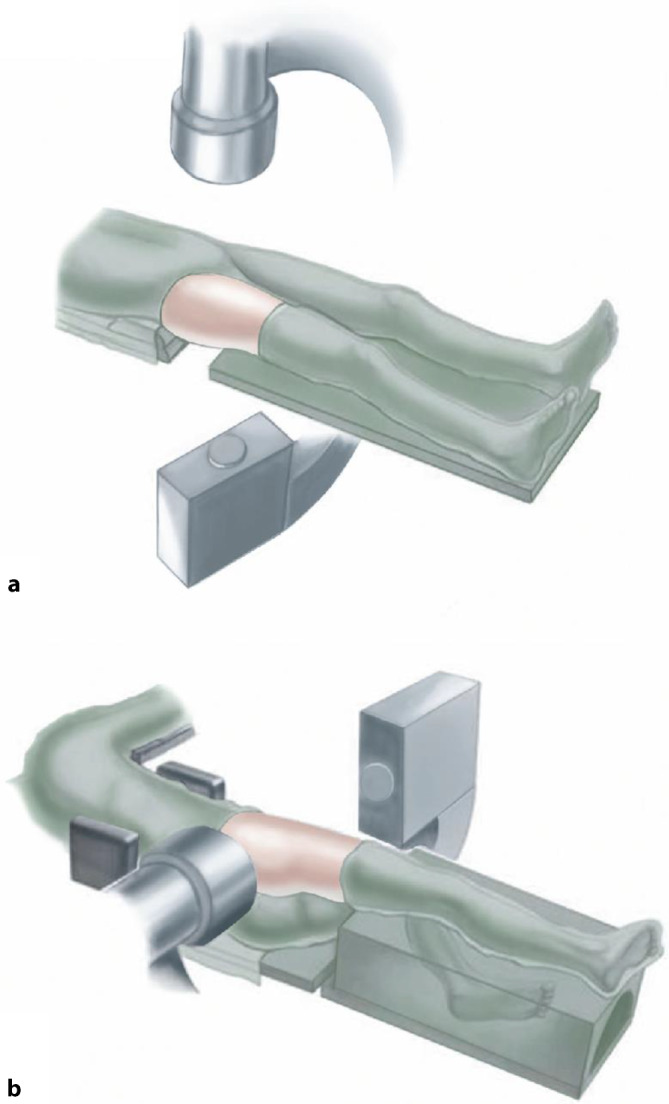

- Intubationsnarkose mit Muskelrelaxation
- Es gibt 2 Lagerungsoptionen:
  - *Rückenlage:* Auf einem röntgenstrahlendurchlässigen Tisch (z. B. Carbontisch) den Körper knapp an den Tischrand und die Füße ganz am Tischende lagern. Anhebung des Gesäßes der zu operierenden Seite durch Unterlegen eines Keillagerungskissens, um die Präparation und die Darstellung zu vereinfachen.
  - *Seitenlage*: Dabei wird der Patient an Rücken und Bauch mit Seitenstützen fixiert oder auf eine Vakuummatratze gelegt. Lagerung des Arms auf Armbank, sodass kein Zug an der Schulter und am Plexus brachialis entsteht. Anwinkelung des unten liegenden Beins. Lagerung des oben liegenden Beins auf gepolstertem, stabilem Block. Sterile und frei bewegliche Abdeckung des ganzen Beins bis zum Beckenkamm. Eine Strahlendurchlässigkeit des Operationstisches ist in Seitenlage nicht notwendig, da der Strahlengang über dem Tisch verläuft.
- Desinfektion des ganzen Beines vom Fuß bis auf die Höhe der Crista iliaca, Abdeckung des Operationsfeldes mit freiliegender Spina iliaca anterior superior und zentriert auf die Schnittführung. Das Bein muss frei beweglich in jeglicher Richtung sein.
- Der Bildverstärker ist so eingestellt, dass eine freie Durchleuchtung (anteroposterior wie auch axial) problemlos möglich ist. Für die a.-p.-Projektion in Rückenlage vertikal, in Seitenlage horizontal (Abb. 4).

## Operationstechnik

Abb. 5, 6, 7, 8, 9, 10, 11, 12, 13, 14, 15, 16, 17.

**Figure 5:**
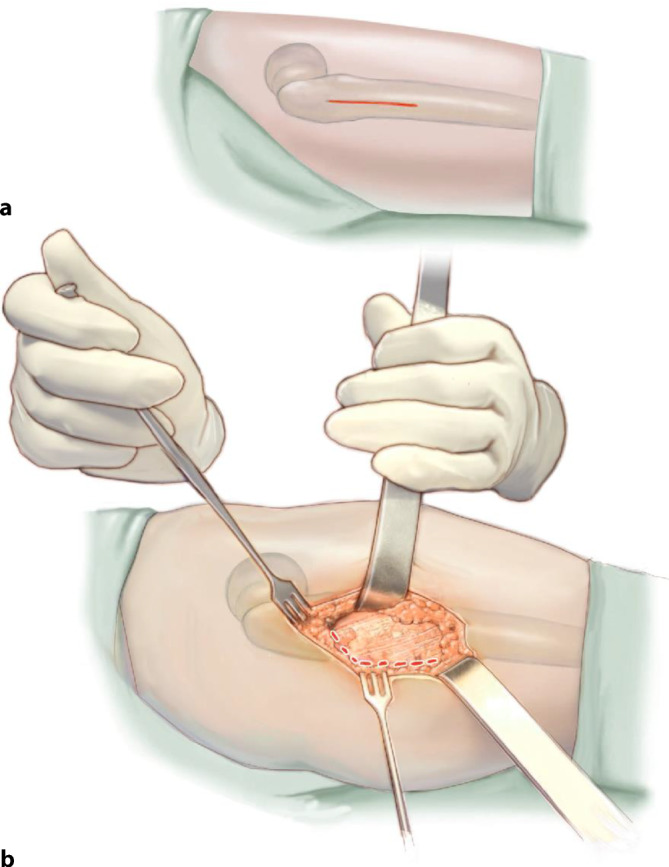

**Figure 6:**
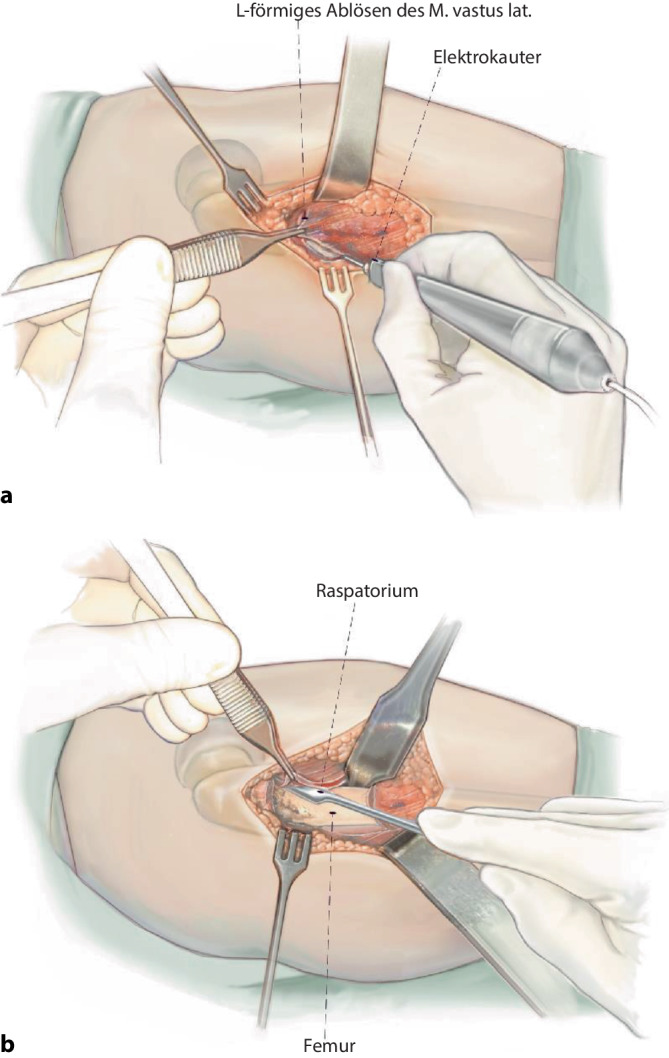

**Figure 7:**
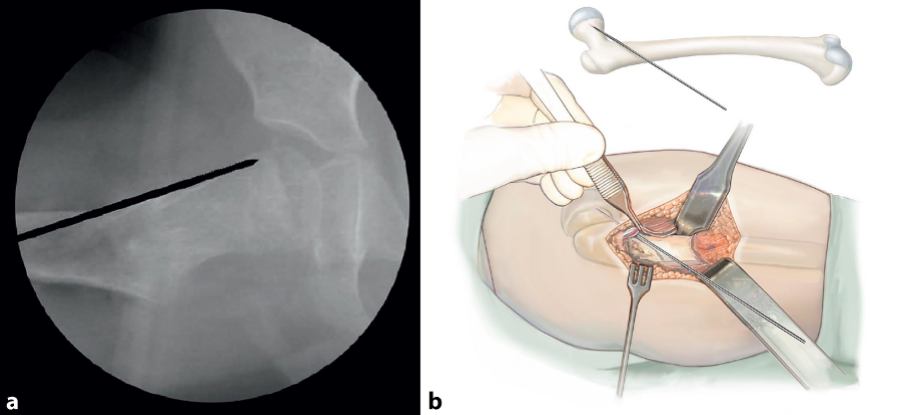

**Figure 8:**
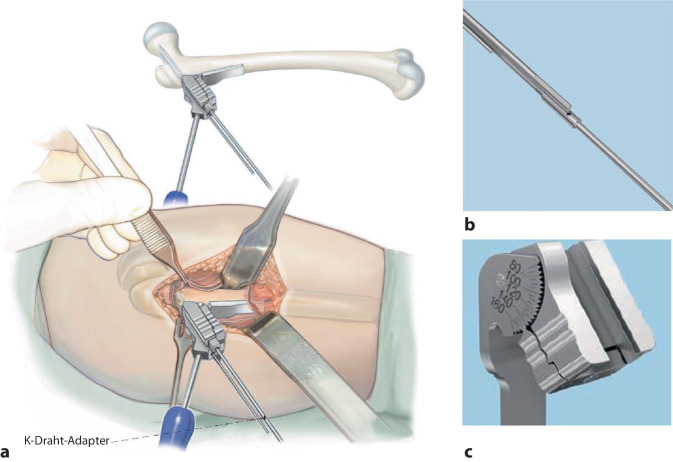

**Figure 9:**
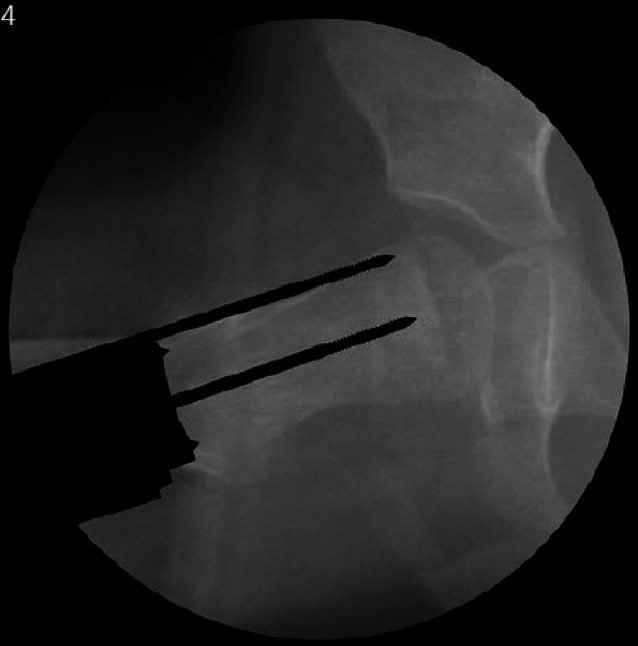

**Figure 10:**
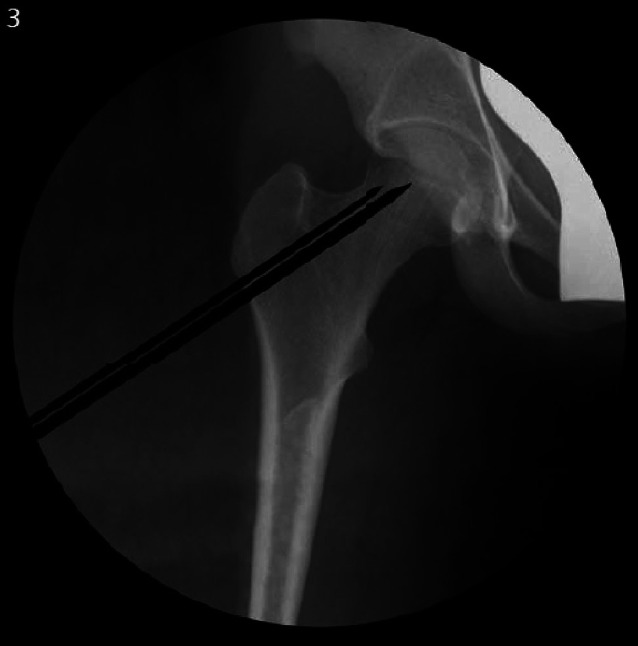

**Figure 11:**
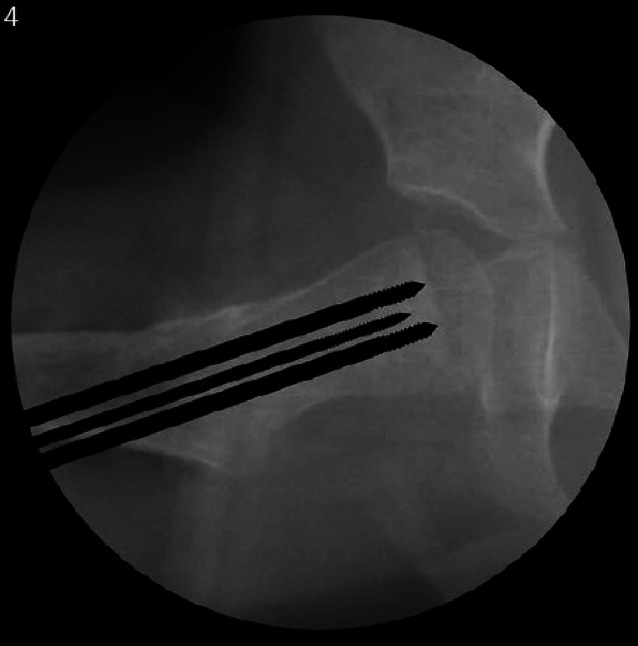

**Figure 12:**
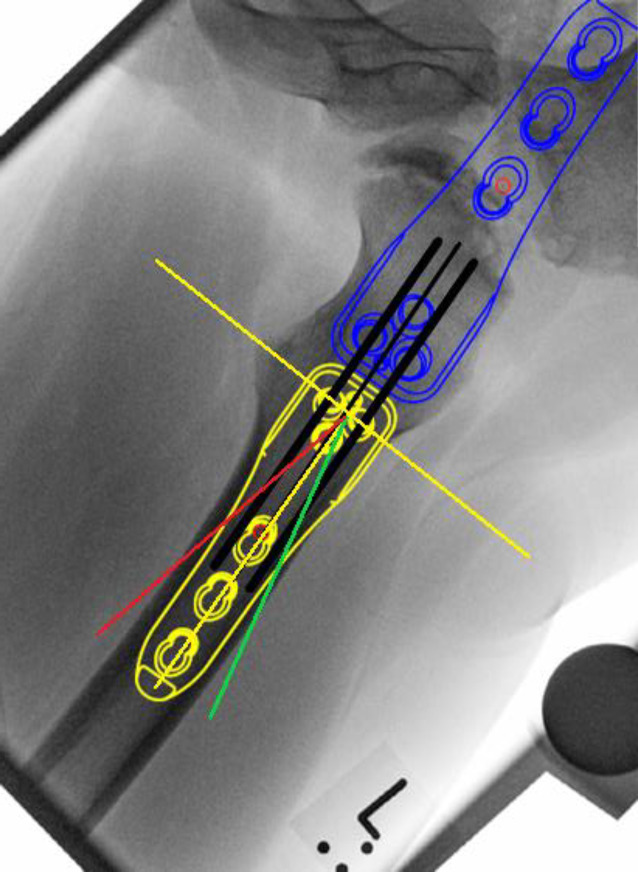

**Figure 13:**
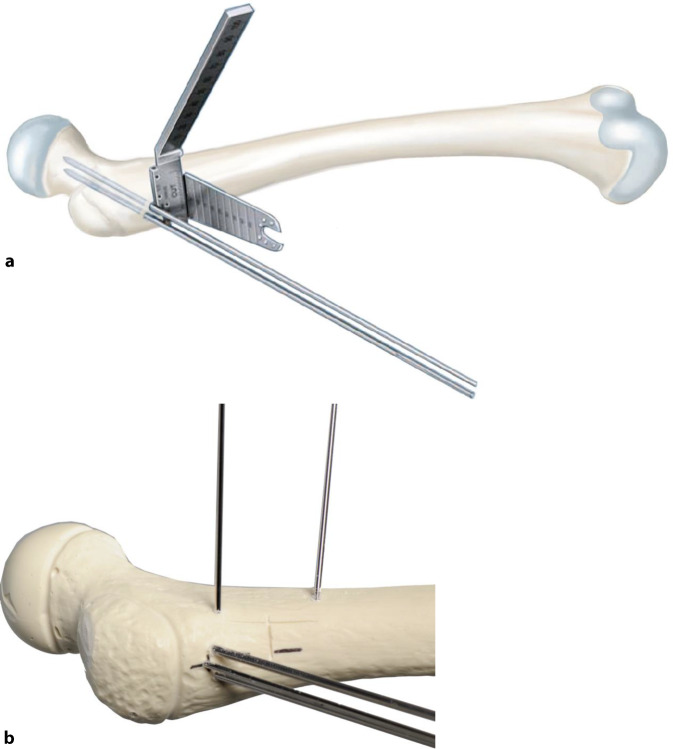

**Figure 14:**
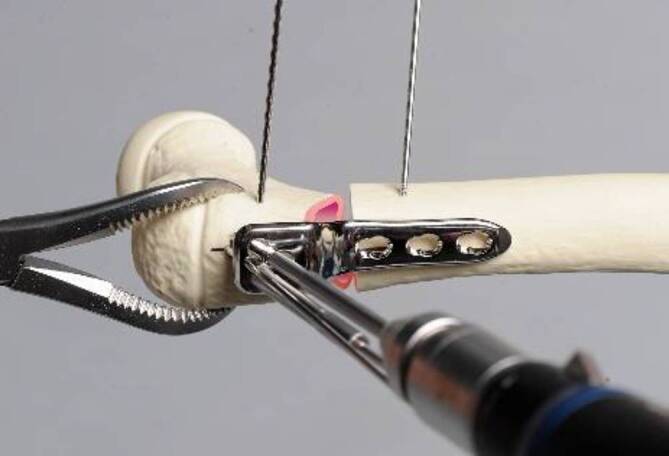

**Figure 15:**
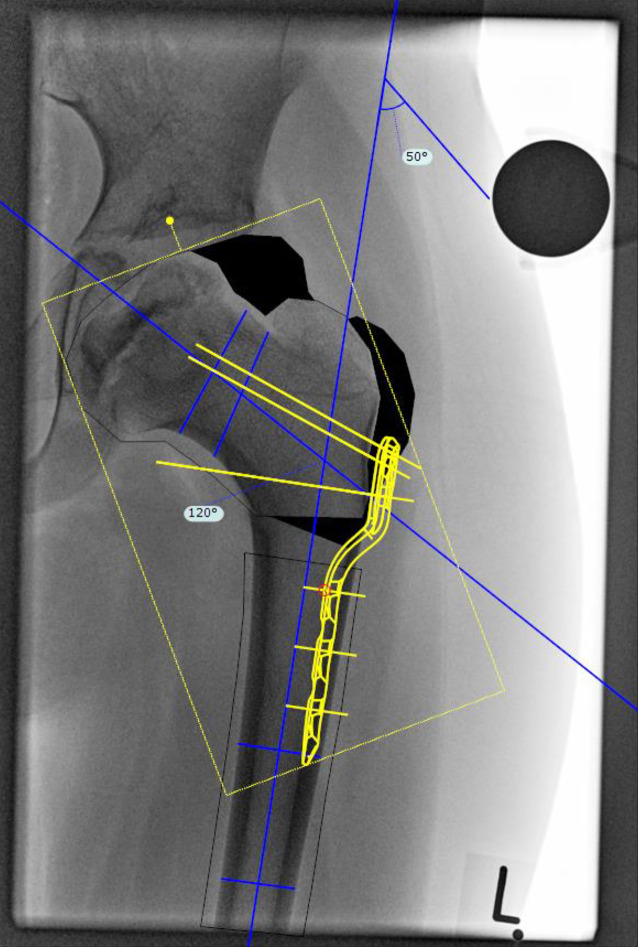

**Figure 16:**
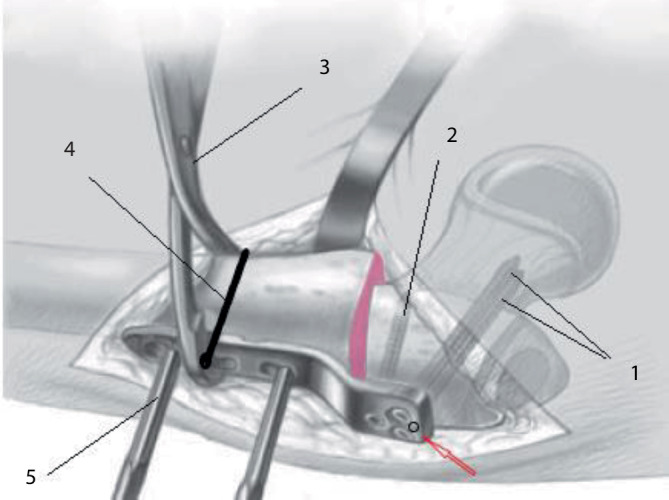

**Figure 17:**
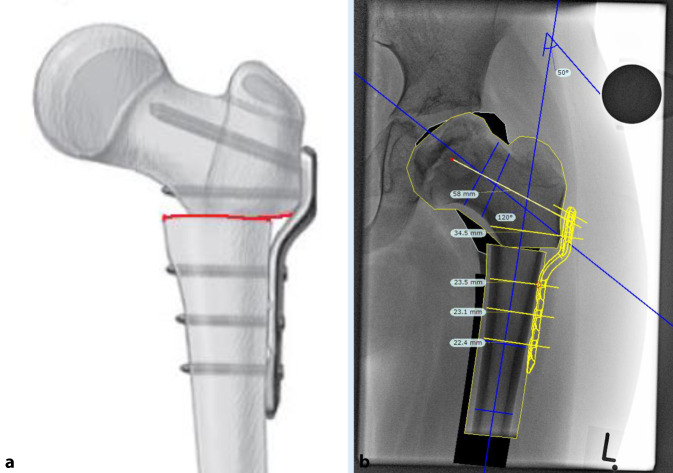

## Besonderheiten

### Medialisation des Femurschaftes

Abb. 18.

**Figure 18:**
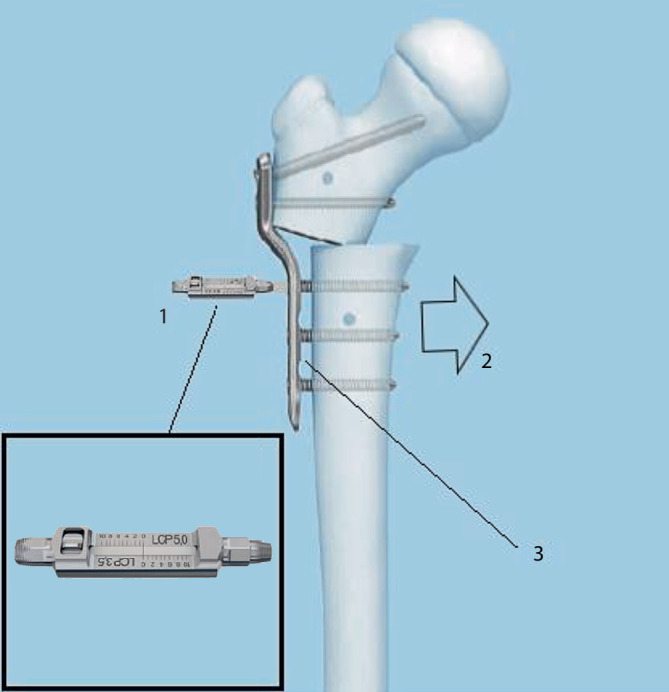

### Alternative Operationstechnik mittels Winkelplatte am Beispiel CAPOS/Fa. DePuy Synthes

**Figure 19:**
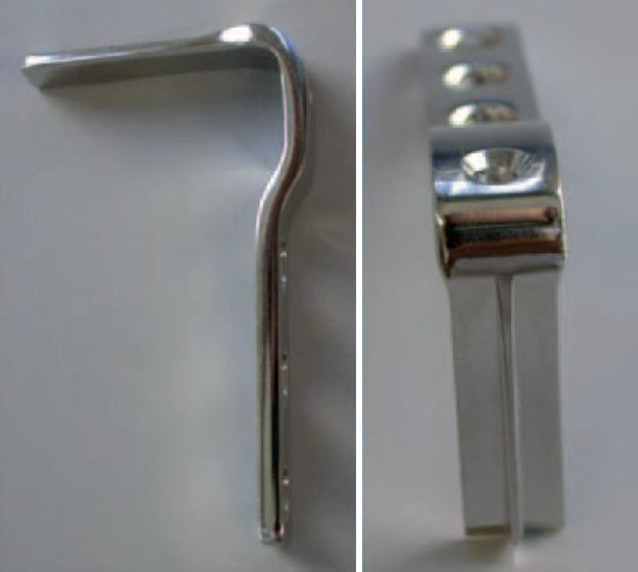

**Figure 20:**
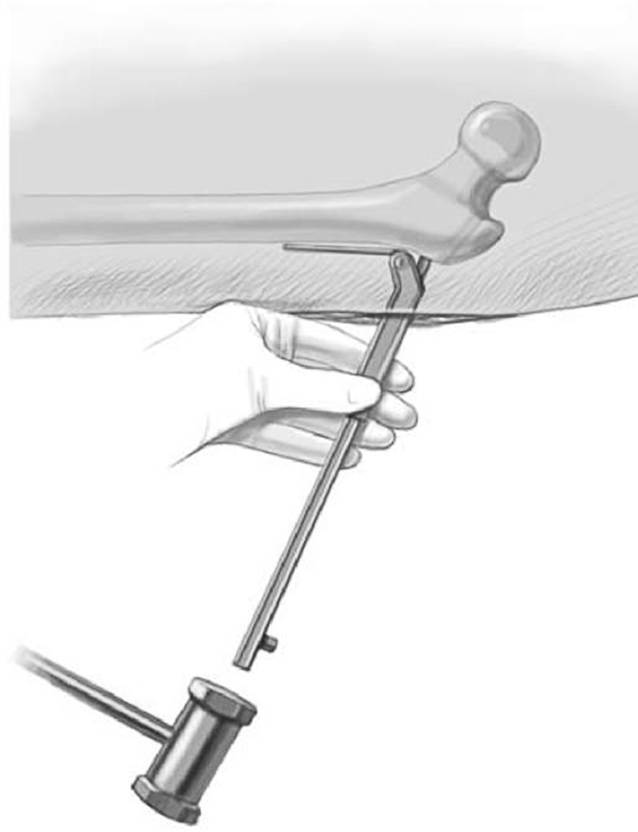

- Hautinzision, Zugang zum proximalen Femur sowie Lokalisation und Markierung der Trochanterapophyse sind ebenfalls gleich. Ebenso sind die Berechnungen vergleichbar, wobei der Platzierungswinkel der LCP-Schrauben hier dem Einschlagwinkel der Plattenklinge entspricht (Abb. 19).
- Die künftige Plattenposition hängt von der genauen Platzierung eines orientierenden Kirschner-Führungsdrahts ab, welcher wie oben beschrieben auch hier axial zentral und parallel zu dem Antetorsionsdraht liegen sollte. Der Einführungspunkt des Plattensetzinstruments liegt ca. 10 mm unterhalb der Trochanterfuge (Abb. 20 und 21).
- Die Osteotomie wird entsprechend Planung ausgeführt und schließlich wird das Plattensetzinstrument durch die definitive Platte ersetzt.

**Figure 21:**
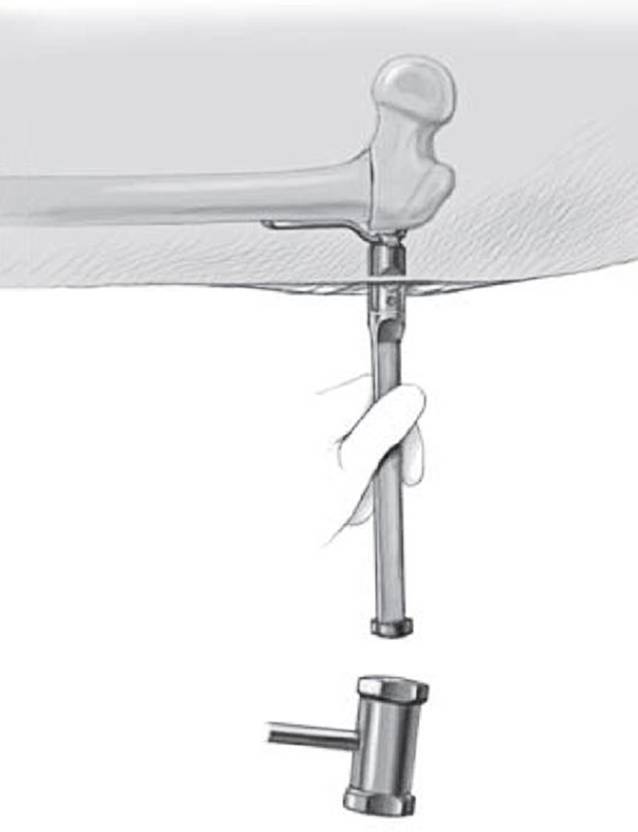

## Postoperative Behandlung

- Bettruhe 1 bis 2 Tage nach der Operation bis zur Schmerzkompensation
- Abrollbelastung an 2 Gehstöcken über mindestens 6 Wochen
- Röntgenkontrolle 6 Wochen nach der Operation und sukzessive Steigerung der Belastung je nach Konsolidation der Osteotomie
- Klinische und radiologische Kontrollen alle 3 Monate bis zur Konsolidation, anschließend alle 6 Monate ausreichend in Bezug auf die Ausheilung des Kopfes
- Implantatentfernung nach 9 bis 12 Monaten
- Rückkehr zum Sport im Hinblick auf Platte und Konsolidation meist nach 3 Monaten möglich; die Grunderkrankung muss hierbei jedoch auch berücksichtigt werden.

## Fehler, Gefahren, Komplikationen

### Verbunden mit chirurgischem Zugang

#### Anterior

- Verletzung des N. femoralis (am lateralsten verlaufende Struktur im ventralen neurovaskulären Bündel) und der Vasa femoralia (medial) bei falsch gesetztem Hohmann-Haken anterior am Femur. Deshalb wird empfohlen, mit dem Haken strikt am Knochen bzw. subperiostal zu bleiben.
- Verletzung von Ästen der A. circumflexa lateralis bei L‑Inzision des M. vastus lateralis, welche zur profusen Blutung führen kann.

#### Posterior

- Verletzung von perforierenden Arterien von dorsal und konsekutive hartnäckige Blutung durch ihre Retraktion

### Verbunden mit der Operationstechnik

- Inkorrekte präoperative Planung und falsche Wahl des Winkels für den Positionierungsdraht führen zur Fehlfixation der Osteotomie.
- Insuffiziente Fixation der Schrauben führt zum Osteosynthesenmaterialversagen und zum Korrekturverlust.
- Durch eine falsche Technik kommt es zur Verletzung von Wachstumsfugen entweder am Trochanter major oder am Femurkopf mit konsekutiver Wachstumsstörung später.
- Infektion von Operationswunde und/oder Implantatlager

## Ergebnisse

Die Resultate der PVO bei Patienten mit LCPD wurden in mehreren Studien untersucht. Unbehandelte Kontrollgruppen fehlen meist oder beinhalten keine vergleichbaren Patientenpopulationen. Diese Tatsache ermöglicht keine belastbare Prognose. Beer berichtete 2008 über eine Patientengruppe von 40 Kindern und 43 operierten Hüftgelenken. Bei einem durchschnittlichen Follow-up (DFU) von 33 Jahren wiesen 24 Hüftgelenke klinisch im „Harris-hip-score“ (HHS) sehr gute und 7 mäßige („fair“) Resultate auf. In dieser Kohorte zeigten 56.1 % eine Ausheilung im Grad I und II nach Stulberg (Grad IV 22 %, Grad V 2.4 %). Nur ein Hüftgelenk zeigte radiologisch eine Koxarthrose der Stufe III nach Tönnis [23]. Im Folgejahr wurden in Korea weitere Daten veröffentlicht. Bei einem kurzen DFU nach Wachstumsabschluss lag bei 60 % dieser LCPD-Hüften ein Stulberg Grad I und II vor, Nur 3 % entfielen auf Grad IV. In dieser Kohorte fand sich keine Hüfte mit Grad V [24]. Weitere 37 Patienten wurden bei einer Studie mit DFU von 42,5 Jahren und Durchschnittsalter von 50,2 Jahren zwecks klinisch-radiologischer Untersuchung eingeschlossen. Bezüglich des HHS waren 64 % im guten oder exzellenten Bereich. Mehr als die Hälfte hatte radiologisch keine oder diskrete Hinweise auf Arthrose. Sieben Patienten hatten bereits eine Hüftprothese bei symptomatischer Koxarthrose erhalten [25]. In einer im Hinblick auf das Follow-up vergleichbaren Studie mit Beobachtung des natürlichen Verlaufs, ohne operative Intervention im Kindesalter, waren von 37 nachkontrollierten Patienten nach 47,7 Jahren DFU bereits 40 % prothetisch behandelt worden [26]. Die Diagnosestellung bei dieser zuletzt erwähnten Kohorte lag jedoch deutlich länger zurück als bei den zuvor erwähnten Studien mit operativer Intervention, so dass auch ein Wandel im Hinblick auf die konservative Behandlung bei der Wertung der Ergebnisse berücksichtigt werden muss. In Bezug auf die Implantate der neuen Generation (LCP-paediatric-hip-plate-System) gibt es wenige retrospektive Studien von pädiatrischen Patienten mit zudem verschiedensten Diagnosen wie Zerebralparese, Hüftdysplasie, LCPD, Frakturen. Ihre Resultate weisen nur geringfügige Komplikationsraten postoperativ auf. Sowohl die implantatbezogenen Komplikationen (Verlust der Korrektur, Lockerung) wie auch eine Beeinträchtigung der Knochenkonsolidation kommen äußerst selten vor [27–29].

PVO ist eine seit mehreren Jahrzenten angewandte und etablierte chirurgische Behandlung von Patienten, welche an schwerem LCPD leiden. Mit der Operation gelingt die Modifikation von intraartikulären (Rezentrierung des Femurkopfes in das Acetabulum) und extraartikulären Stellungsverhältnissen (Flexions‑, Extensionsosteotomie, Derotation zwecks Entlastung der Nekrosenzone). Der Stellenwert dieser etablierten Technik ergibt sich weniger aus einer umfangreichen Studienlage als vielmehr aus bereits langfristiger Erfahrung mit dieser Therapie seitens der behandelnden orthopädischen Chirurgen.
